# Evaluation of hemostasis in hyperthyroid cats

**DOI:** 10.1111/jvim.16274

**Published:** 2021-09-30

**Authors:** Audrey E. Keebaugh, Stefanie M. DeMonaco, David L. Panciera, Jonathan A. Abbott, Katie M. Boes, Giulio Menciotti

**Affiliations:** ^1^ Department of Small Animal Clinical Sciences Virginia‐Maryland College of Veterinary Medicine Blacksburg Virginia USA; ^2^ Department of Small Animal Clinical Sciences, College of Veterinary Medicine University of Tennessee Knoxville Tennessee USA; ^3^ Department of Biomedical Sciences & Pathobiology Virginia‐Maryland College of Veterinary Medicine Blacksburg Virginia USA

**Keywords:** coagulation, hypercoagulability, radioiodine, thromboembolism

## Abstract

**Background:**

Hyperthyroid cats might have a predisposition to arterial thrombus formation. The mechanism for thrombogenesis currently is unknown but could be associated with systemic hypercoagulability as seen in hyperthyroid humans.

**Objective:**

Our purpose was to evaluate markers of hemostasis in hyperthyroid cats compared to healthy cats, and in hyperthyroid cats before and after radioactive iodine treatment (RIT).

**Animals:**

Twenty‐five cats with hyperthyroidism and 13 healthy euthyroid cats >8 years of age.

**Methods:**

Prothrombin time (PT), activated partial thromboplastin time (aPTT), fibrinogen concentration, antithrombin (AT), D‐dimers, thrombin‐antithrombin complexes (TAT), von Willebrand Factor antigen (vWF : Ag), and activity of factors VIII and IX were measured. An echocardiogram was performed in all cats. Hemostatic markers and echocardiogram were evaluated again 6 to 9 months after successful RIT in 7 cats.

**Results:**

Hyperthyroid cats had higher fibrinogen concentration (*P* < .0001), AT activity (*P* < .0001), and vWF : Ag concentration (*P* = .01) than healthy control cats with all results decreasing significantly post‐RIT. Hyperthyroid cats were not more likely to be in a hypercoaguable state than euthyroid cats (*P* = .08). Serum T4 concentration was not a predictor of a hypercoagulable state (*P* = .53).

**Conclusions and Clinical Importance:**

Hyperthyroid cats have evidence of altered hemostasis that does not appear to be solely attributable to cardiac abnormalities, but no evidence of a hypercoagulable state. Findings suggest altered hemostasis resolves after RIT. Hyperthyroid cats could have endothelial dysfunction as indicated by increased vWF : Ag which could potentiate thrombogenesis.

**Abbreviations**
aPTT: activated partial thromboplastin time
AT: antithrombin
ATE: arterial thromboembolism
LA : Ao: left atrium to aorta ratio
PT: prothrombin time
RIT: radioactive iodine treatment
RI: reference interval
TAT: thrombin‐antithrombin complexes
TE: thromboembolism
vWF: von Willebrand factor
vWF : Ag: von Willebrand factor antigen

## 1 INTRODUCTION

In humans, hyperthyroidism alters the coagulation‐fibrinolytic balance creating a hypercoagulable state that increases the risk of thromboembolism (TE).^1^ The risk of venous thrombosis increases with higher serum concentrations of free thyroxine in people.^2^ The mechanisms by which hyperthyroidism induces a hypercoagulable state in humans are thought to involve all stages of hemostasis. Primary hemostasis is enhanced by increased circulating von Willebrand Factor (vWF) concentration, which promotes platelet adhesion to the subendothelium.^3^, ^4^ Hyperthyroidism also induces increased secondary hemostatic function by increases in coagulation proteins such as factor VIII, factor IX, and fibrinogen.^3^, ^4^ In addition, fibrinolysis is impaired as a result of increased plasminogen activator inhibitor concentration.^3^, ^4^ Finally, hyperthyroidism also leads to endothelial dysfunction, which contributes to a hypercoagulable state in people.^4^

Similar to hyperthyroid humans, cats with hyperthyroidism might be in a hypercoagulable state, but the mechanism of potential thrombus formation in hyperthyroid cats is unknown. The patterns of thrombosis in the 2 species are different. Venous thrombosis is described in hyperthyroid people, whereas arterial thrombosis is most common in cats.^1^, ^5^ Mechanisms of thrombus formation are multifactorial and include abnormal blood flow, endothelial damage, and hypercoagulability.^6^ In cats with cardiomyopathy, thrombus formation has been attributed to blood stasis and endothelial injury.^7^, ^8^ Some evidence suggests that cats with cardiomyopathy also might be hypercoagulable.^8^, ^9^, ^10^, ^11^ Hyperthyroidism results in complex changes that alter circulation and cardiac structure and function.^11^ The resultant thyrotoxic cardiac disease could contribute to thrombus formation in hyperthyroid cats.^12^, ^13^ However, a hyperthyroid cat with an echocardiographically normal heart has been documented to have had an episode of arterial thromboembolism (ATE).^5^ A predisposition for thrombus formation can exist independent of structural abnormalities such as atrial enlargement.^10^ This possibility is consistent with the existence of mechanisms other than cardiac disease that might be responsible for TE in hyperthyroid cats.^5^ Hyperthyroid cats have been documented to have changes consistent with a hypercoagulable state as indicated by hyperfibrinogenemia and shortened PT, which might explain the occurrence of TE in affected cats that do not have clinically detectable thyrotoxic heart disease.^14^

The purpose of our study is to evaluate hemostatic markers in hyperthyroid cats as compared to healthy controls and after treatment with RIT. Our hypotheses were that hyperthyroid cats would have altered hemostatic markers and that altered hemostasis would resolve after successful RIT. We also hypothesized evidence of systemic hypercoagulability would be found.

## 2 MATERIALS AND METHODS

### 2.1 Animals

Client‐owned cats referred for radioiodine treatment (RIT) of hyperthyroidism were eligible for enrollment. Hyperthyroidism was defined as a serum T4 concentration that exceeded the upper limit of the reference interval (ie, T4 > 37.7 nmol/L) as well as at least 1 compatible finding on history (eg, polyphagia, weight loss, polyuria, polydipsia, hyperactivity) and 1 compatible finding on physical examination (eg, presence of thyroid nodule, poor body, and muscle condition scores). Cats were excluded if they had received methimazole or a low‐iodine diet in the 2 weeks before presentation. In addition, cats were excluded if they had received medications known to alter hemostatic variables (eg, prednisone, aspirin, clopidogrel, heparin) or had evidence of concurrent illness. Staff‐ and student‐owned cats deemed healthy based on routine physical examination, a CBC, serum or plasma biochemistry, serum T4 concentration, urinalysis, and echocardiography made up the control group. Cats in both groups were given a standardized sedation protocol IM (alfaxalone, 2 mg/kg and butorphanol, 0.2 mg/kg) if temperament prohibited evaluation.

### 2.2 Hemostatic markers

Approximately 2.7 mL of blood was collected into a blood collection tube (BD Vacutainer, Becton, Dickinson and Company, Franklin Lakes, New Jersey) containing 0.3 mL citrate by jugular venipuncture with a butterfly needle and allowing the vacuum to draw in the blood sample with 4 complete inversions of the blood collection tube postcollection. Blood samples were not analyzed if citrated samples contained clots or if venipuncture was traumatic. Blood was transferred into a polypropylene tube and centrifuged for 10 minutes at 5°C and 1500*g*. Plasma was transferred to a polyethylene tube and stored at −70°C until shipped for batch analysis (samples sent approximately every 4‐6 months) to the Comparative Coagulation Laboratory at Cornell University College of Veterinary Medicine. Plasma was analyzed for prothrombin time (PT), activated partial thromboplastin time (aPTT), concentrations of fibrinogen, D‐dimers, antithrombin (AT), thrombin‐antithrombin complexes (TAT), von Willebrand factor antigen (vWF : Ag), and activity of factors VIII and IX.^8^ Hypercoagulability was defined as ≥2 of the following abnormalities in hyperthyroid cats relative to reference intervals: decreased AT activity, increased factor VIII or IX activities, or increased concentrations of TAT, fibrinogen, or D‐dimers.^8^ Hyperthyroid cats were grouped based on the presence or absence of structural cardiac abnormalities to compare markers of hemostasis, and thus determine if cardiac characteristics might contribute to alterations of hemostasis. When possible in hyperthyroid cats, markers of hypercoagulability again were analyzed 6 to 9 months after RIT if resolution of hyperthyroidism, defined by a serum T4 concentration within the reference interval, was documented.

### 2.3 Echocardiography

Complete echocardiograms were performed at the time of enrollment in all cats by a single operator (GM).^15^ Echocardiograms were performed with an ultrasound unit (Aplio i900, Canon Medical Systems USA, Tustin, California) equipped with a pediatric transducer (PSI‐70BT, Toshiba Medical Systems, Tokyo, Japan). All measurements were performed off‐line by a single operator (JA), blinded to the cat's thyroid status. End‐diastolic left ventricular dimensions were measured on M‐mode images obtained with 2‐dimensional (2D) guidance from a right parasternal short‐axis view of the left ventricle at the level of the papillary muscles. End‐diastole was defined as the onset of the QRS complex on the concomitantly acquired ECG tracing, and measurements were performed using “leading edge‐to‐leading edge” technique. Additionally, right parasternal long‐axis images in which the outflow tract was visible were analyzed. End‐diastolic frames were identified as the first frame of closure of the mitral valve. In these frames, the thickest segment of the interventricular septum was measured using “leading edge‐to‐trailing edge” technique, perpendicular to the endocardial borders; measurements of the left ventricular (LV) lumen were made perpendicular to the long‐axis of the ventricle at the thickest region and excluded the endocardium; and, the thickest portion of the posterior wall was measured using “leading edge‐to‐leading edge” technique, perpendicular to the endocardial border and the pericardium. Papillary muscles were excluded from all measurements. From short‐axis images, left atrial and aortic dimensions were obtained from the first diastolic frame in which closure of the aortic valve was evident. The aortic dimension was measured parallel to the commissure of the non‐ and right‐coronary aortic valve cusps. The left atrial dimension was measured parallel to the commissure of the left‐ and noncoronary aortic valve cusps. The left atrial : aortic ratio was derived from these dimensions. Cats with measurements of left ventricular free wall and interventricular septal wall thickness at end‐diastole <6 mm in any of the abovementioned measurements, left atrium‐to‐aorta ratio (LA : Ao) ≤ 1.5, no or only trivial (clinically irrelevant) insufficiencies of the pulmonic and tricuspid valves, and no insufficiency of the aortic and mitral valves comprised the control group. Cats screened for inclusion as healthy controls were excluded if echocardiographic abnormalities were noted. Hyperthyroid cats with echocardiographic abnormalities remained in the study and were placed in the abnormal echocardiogram group.

Hyperthyroid cats received RIT by SC route, with dosing based on the patient's serum T4 concentration and thyroid scintigraphy results and, if documented to have resolution of hyperthyroidism as defined by a serum T4 concentration within the reference interval 6 to 9 months after treatment, had an echocardiogram performed identical to that before treatment. All echocardiograms were performed by the same operator and measurements were made by an observer blinded to the status of the cat as described above.

### 2.4 Statistical analysis

Statistical analyses were performed by commercially available computer software (SAS Version 9.4, Cary, North Carolina). A power analysis based on previous findings showed that 13 cats per group (hyperthyroid and healthy controls) would be needed to detect a difference of 0.40 in the proportions of cats in a hypercoagulable state (defined below) with a power of 82.8%.^8^

The proportion of cats with a hypercoagulable state was compared between the 2 groups (hyperthyroid and control) by Fisher's exact test. Predications for the prevalence of hypercoagulability were made based on a previous study evaluating hypercoagulability in cats with cardiac disease.^8^ For continuous variables, normal probability plots were used to determine whether the distribution of the data was approximately normal. Normal probability plots showed that all coagulation variables including PT, aPTT, fibrinogen, AT, D‐dimers, vWF : Ag, TAT, factor VIII, and factor IX were not normally distributed. The corresponding data were summarized as medians (range). The Wilcoxon rank sum test then was used to compare coagulation variables (1 at a time) between groups of cats defined as follows: (a) initial hyperthyroid cats versus controls; (b) initial hyperthyroid cats with normal echocardiograms versus cats with abnormal echocardiograms; and (c) cats that required sedation for blood collection versus cats that did not require sedation for blood collection.

A logistic model was used to define the association between both serum T4 concentration and left atrial size to the binary response variable, hypercoagulability [yes/no]. Spearman correlation and linear regression analyses were used to test the association between T4 and the hemostatic variables showing a difference before and after treatment in hyperthyroid cats.

An alpha value of .05 was set for all tests.

## 3 RESULTS

### 3.1 Study population

Twenty‐five hyperthyroid cats met the inclusion criteria and were enrolled in the study. The median age of the 25 cats in the hyperthyroid group (13 years; range, 10‐16 years) was not different from the 13 cats in the control group (12 years; range, 8‐16 years). The hyperthyroid group consisted of 13 spayed females and 12 neutered males and the euthyroid group was composed of 9 spayed females and 4 neutered males. Breeds from both groups included domestic longhair and shorthair (34 cats), Himalayan (2 cats), Siamese (1 cat), and Burmese (1 cat). Median body weight of the hyperthyroid cats was 4 kg (range, 2‐6.3 kg) and the median body weight of the euthyroid controls was 4.9 kg (range, 3.1‐6.4 kg). Sixteen cats in the hyperthyroid group had received methimazole either PO or transdermally, with median treatment duration of 4 months (range, 2 weeks to 35 months). For these 16 cats that previously had received methimazole, the median dose was 2.23 mg/kg/day (range, 0.76‐5.05 mg/kg/day). No other treatment for hyperthyroidism was noted (eg, iodine‐restricted diet, thyroidectomy, previous RIT). In the hyperthyroid group, 18 of the 25 cats required sedation for echocardiographic evaluation and 19 cats required sedation for jugular venipuncture. In the control group, 7 of the 13 cats required sedation for both echocardiographic evaluation and jugular venipuncture.

Nine cats in the hyperthyroid group underwent a second evaluation at a median of 6 months post‐RIT (range, 6‐9 months). Ten of the initial hyperthyroid cats were lost to follow‐up, 3 were euthanized before the reevaluation time frame, 2 were hyperthyroid based on serum T4 concentration at 6 months post‐RIT, and 1 did not receive RIT.

### 3.2 Serum T4 concentrations

The median serum T4 concentration in the hyperthyroid cats was 130 nmol/L (reference interval [RI], 16.0‐37.7 nmol/L; range, 49‐509 nmol/L). The median serum T4 concentration in the euthyroid control cats was 28.4 nmol/L (range, 19.4‐34.9 nmol/L).

Of the 9 cats evaluated post‐RIT, 2 were persistently hyperthyroid and were excluded from analysis. Serum T4 concentration was normal in 7 cats (median, 18.4 nmol/L; range, 16.3‐33.5 nmol/L), and the median (range) serum thyroid‐stimulating hormone (TSH) concentration was 0.076 (0.02‐2.87) ng/mL. Two of the 7 cats had serum TSH concentrations >0.3 ng/mL.

### 3.3 Echocardiography

**FIGURE 1:**
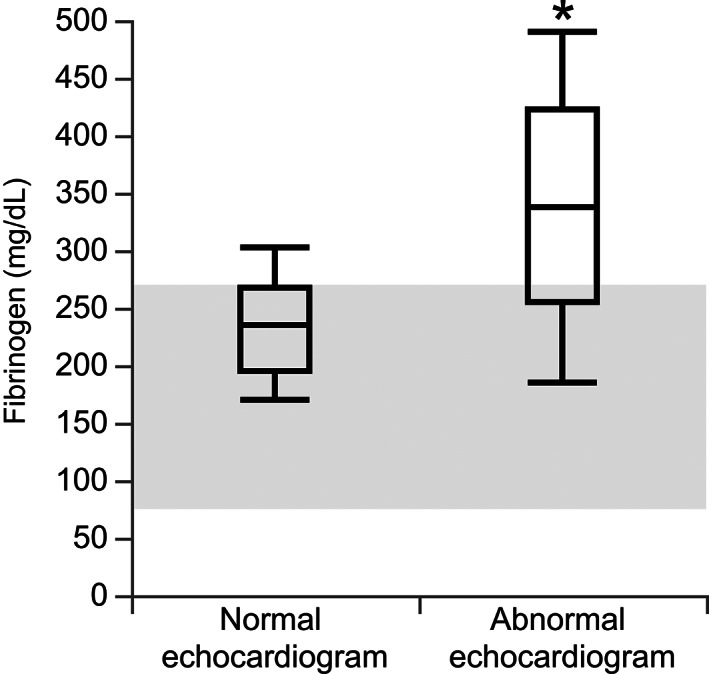
Boxplots of plasma fibrinogen concentrations in 6 hyperthyroid cats with normal echocardiograms and 19 hyperthyroid cats with abnormal echocardiograms. Boxes represent the interquartile range (IQR) from the 25th to 75th percentile. The horizontal bar in each box represents the median value. The whiskers indicate the range of values. The shaded area indicated the reference interval. *P = .02

Based on the established criteria, 19/25 (76%) hyperthyroid cats had abnormal echocardiographic findings (Table 1). Of the 19 hyperthyroid cats that had abnormal echocardiographic findings, 13 cats had been treated previously with methimazole and 3 of these cats reportedly had their hyperthyroidism controlled on methimazole. Plasma fibrinogen concentrations of hyperthyroid cats that were echocardiographically abnormal (median, 337 mg/dL; range, 185‐490 mg/dL) were significantly higher than fibrinogen concentrations of hyperthyroid cats that were echocardiographically normal (median, 235 mg/dL; range, 171‐303 mg/dL; *P* = .02; Figure 1). The presence or absence of echocardiographic abnormalities in hyperthyroid cats did not have a significant effect on the other measured hemostatic markers (Table 2). We did not identify a linear association between serum T4 concentration and LA : Ao ratio.

**TABLE 1:** Echocardiographic data of hyperthyroid cats (n = 25)

Measurement	Median	25th‐75th quartile	Number of abnormal
LVPWd 2D	4.8 mm	4.3‐5.4 mm	3 (12%)
LVPWd M‐mode	5.5 mm	5.2‐6.1 mm	8 (32%)
IVSd 2D	5.6 mm	4.9‐6.8 mm	11 (44%)
IVSd M‐mode	5.4 mm	4.4‐5.9 mm	6 (24%)
LA : Ao 2D	1.22	1.07‐1.38	2 (8%)

Abbreviations: LVPWd, left ventricular posterior wall end diastole; IVSd, interventricular septal end diastole; LA : Ao, left atrium to aorta ratio; 2D, 2‐dimensional.

**TABLE 2:** Markers of hemostasis in hyperthyroid cats with normal and abnormal echocardiograms

Marker of hemostasis	Normal echo (n = 6)	Abnormal echo (n = 19)	Reference intervals	*P*‐value
PT	17.8 (16.9‐18.7)	17.4 (16‐18.7)	15‐20 s	.27
aPTT	14.9 (12‐16.7)	15.8 (12‐30.4)	15‐21 s	.43
Fibrinogen	235 (171‐303)	337 (185‐490)	76‐270 mg/dL	.03
AT	119 (100‐130)	116 (82‐131)	75‐110%	.95
D‐dimer	342 (0‐1411)	139 (0‐676)	0‐250 ng/mL	.38
vWF : Ag	155 (132‐221)	180 (112‐253)	70‐180%	.19
TAT	12.8 (3.8‐124)	5.7 (2.6‐46.1)	1‐8 μg/L	.17
Factor VIII	87 (41‐495)^a^	92 (18‐388)^b^	50‐200%	.67
Factor IX	114 (67‐277)^a^	110 (45‐176)^b^	50‐150%	.8

^a^ Results available for 5 cats.

^b^ Results available for 15 cats.

Seven cats in the hyperthyroid group had echocardiography performed after RIT and documentation of normal serum T4 concentration. Six of the 7 cats had abnormal echocardiograms on initial presentation. Only 1 of these 6 cats had resolution of the echocardiographic changes after treatment. Comparison of LA : Ao obtained before and after treatment did not identify a difference (*P* = .05).

### 3.4 Hemostatic markers

Compared to the RIs established by the Comparative Coagulation Laboratory at Cornell University College of Veterinary Medicine, aPTT was decreased in 9/25 (36%) and 5/13 (38%), fibrinogen was increased in 14/25 (56%) and 0/13 (0%), D‐dimers were increased in 8/25 (32%) and 3/13 (23%), TAT was increased in 10/25 (40%) and 2/13 (15%), factor VIII was increased in 4/20 (20%) and 3/13 (23%), and factor IX was increased in 6/20 (30%) and 4/13 (30%) of hyperthyroid and control cats, respectively.

**FIGURE 2:**
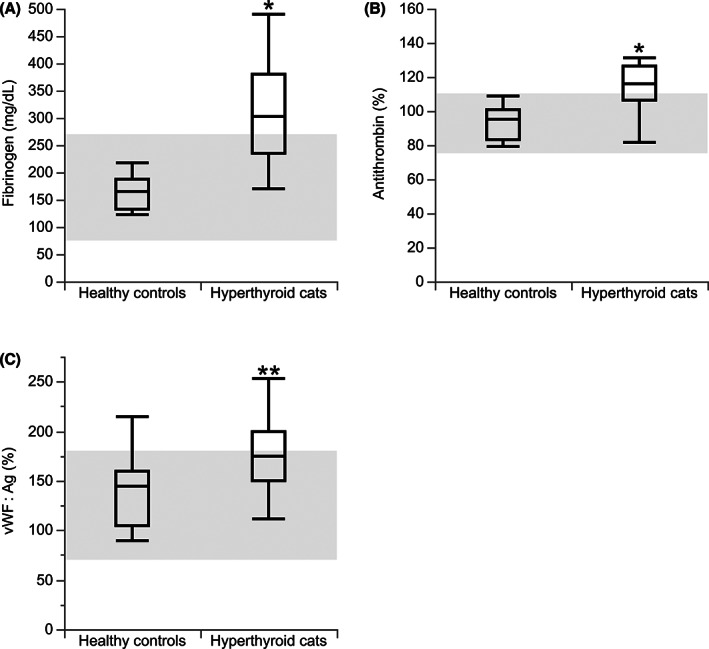
Boxplots of hemostasis markers in 25 cats with hyperthyroidism and 13 euthyroid control cats. (A) Plasma fibrinogen concentration; (B) plasma antithrombin activity; (C) plasma von Willebrand Factor antigen (vWF : Ag) concentration. *P < .0001; **P = .01

**FIGURE 3:**
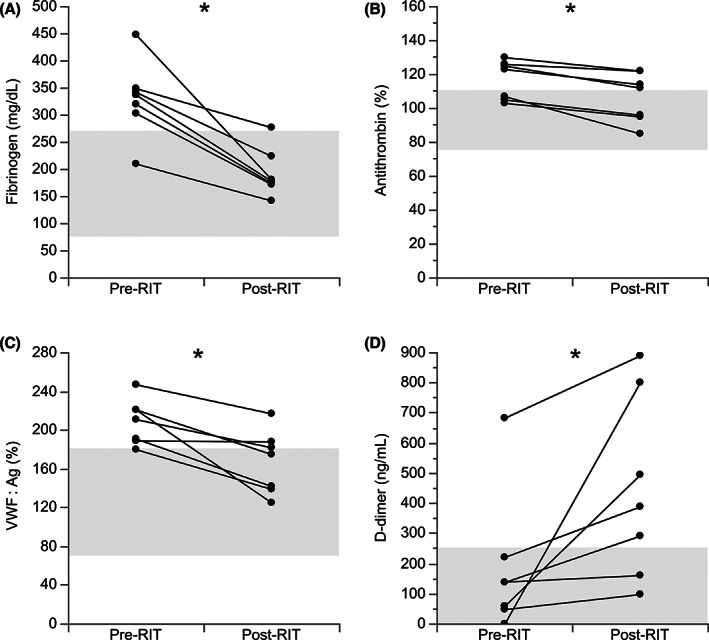
Hemostasis markers in 7 hyperthyroid cats before and after radioactive iodine therapy. (A) Plasma fibrinogen concentration; (B) plasma antithrombin activity; (C) plasma von Willebrand Factor antigen (vWF : Ag) concentration; (D) plasma D‐dimer concentration. *P = .02

Cats with hyperthyroidism had higher plasma fibrinogen concentration than euthyroid controls (*P* < .0001; Figure 2). If hyperthyroidism resolved after RIT, fibrinogen concentration decreased (*P* = .02; Figure 3) when compared with pretreatment concentration. Cats with hyperthyroidism also had higher AT activity than euthyroid controls (*P* < .0001; Figure 2). After RIT, AT activity decreased (*P* = .02; Figure 3) compared to pretreatment. The hyperthyroid cats also had higher vWF : Ag concentrations compared to pretreatment results. The hyperthyroid group also had higher vWF : Ag concentrations compared to euthyroid controls (*P* = .01; Figure 2) that decreased after treatment (Figure 3). No difference was found in D‐dimer concentrations between hyperthyroid cats and euthyroid controls, but after RIT, the D‐dimer concentration increased compared to initial results in hyperthyroid cats (*P* = .02; Figure 3).

For the remaining markers of hemostasis in hyperthyroid cats (PT, aPTT, TAT, factor VIII, factor IX), no differences were found when compared to euthyroid controls or post‐RIT results (Tables 3 and 4).

**TABLE 3:** Markers of hemostasis in hyperthyroid and euthyroid control cats

Marker of hemostasis	Hyperthyroid (n = 25)	Control (n = 13)	Reference intervals	*P*‐value
PT	17.4 (16‐18.7)	17.1 (16‐19)	15‐20 s	.85
aPTT	15.5 (12‐30.4)	15 (13‐19)	15‐21 s	.71
Fibrinogen	303 (171‐490)	164 (123‐218)	76‐270 mg/dL	<.001
AT	116 (82‐131)	95 (79‐109)	75‐110%	<.001
D‐dimer	139 (0‐1411)	131 (48‐1226)	0‐250 ng/mL	.84
vWF : Ag	175 (112‐253)	144 (89‐214)	70‐180%	.01
TAT	6.2 (2.6‐124)	4.8 (2.6‐44.4)	1‐8 μg/L	.46
Factor VIII	89.5 (18‐495)^a^	97 (37‐369)	50‐200%	.96
Factor IX	112.5 (45‐277)^a^	107 (50‐285)	50‐150%	.64

^a^ Results available for 20 cats.

**TABLE 4:** Markers of hemostasis in initial hyperthyroid and euthyroid cats post RIT (n = 7)

Marker of hemostasis	Initial hyperthyroid	Recheck euthyroid	Reference intervals	*P*‐value
PT	17 (16.7‐18.3)	17.7 (17‐18.6)	15‐20 s	.16
aPTT	16.1 (13.5‐18)	16 (14.5‐18.3)	15‐21 s	.81
Fibrinogen	337 (210‐448)	178 (142‐277)	76‐270 mg/dL	.02
AT	123 (103‐130)	112 (85‐122)	75‐110%	.02
D‐dimer	138 (0‐683)	389 (98‐890)	0‐250 ng/mL	.02
vWF : Ag	211 (180‐247)	175 (125‐217)	70‐180%	.02
TAT	10.5 (2.6‐46.1)	11.5 (2‐16.1)	1‐8 μg/L	1
Factor VIII^a^	84 (18‐144)	109.5 (59‐174)	50‐200%	.06
Factor IX^a^	106 (45‐154)	111 (74‐175)	50‐150%	.44

^a^ Results available for 6 cats.

Serum T4 concentration was positively correlated with fibrinogen (*r* = 0.79; 95% confidence interval [CI], 0.63‐0.89; *P* < .0001), AT (*r* =  0.70; 95% CI, 0.50‐0.84; *P* < .0001), and vWF : Ag (*r* = 0.48; 95% CI, 0.18‐0.70; *P* = .003) concentrations. Serum T4 concentration was not correlated with D‐dimer concentration.

Sedation did not have an apparent effect on any hemostatic variable.

### 3.5 Hypercoagulability

Two hyperthyroid cats were excluded from analysis of hypercoagulability because of lack of factor VIII and factor IX measurements, leaving 23 hyperthyroid cats in the analysis. The prevalence of hypercoagulability (as previously defined) was 14/23 (60.9%) in hyperthyroid cats and 4/13 (30.8%) in the control group, although no difference was found in the proportions of hyperthyroid and euthyroid cats that were hypercoagulable (*P* = .08). Also, the increase in serum T4 concentration was not a predictor of hypercoagulability (*P* = .53). When both hyperthyroid and control groups were included, 11/18 (61.1%) of the defined hypercoagulable cats had abnormal echocardiograms.

In the 7 cats that were reassessed after RIT, 5 of those cats on initial evaluation before RIT were classified as hypercoagulable. Hypercoagulability resolved after RIT in 1 of the 5 cats when reevaluated. Four of these 5 cats had abnormal echocardiograms on initial presentation. In the cat that had resolution of its hypercoagulable state, the echocardiographic abnormalities persisted. One cat with an abnormal echocardiogram that was not defined as hypercoagulable on presentation had a normal echocardiogram after RIT and had persistently normal coagulation status. Four cats had persistence of the defined hypercoagulable state and concurrent abnormal echocardiograms.

## 4 DISCUSSION

Our results provide evidence of altered markers of hemostasis in hyperthyroid cats, including evidence of coagulation factor excess, that resolves after RIT, but no evidence of a hypercoagulable state. These alterations do not appear to be solely attributed to cardiac status. Although a hypercoagulable state caused by hyperthyroidism could not be established by the criteria chosen, increases in plasma fibrinogen and vWF : Ag concentrations in the hyperthyroid group were consistent with hypercoagulability. Our definition of hypercoagulability was adopted from a previous study that evaluated hypercoagulability in cats with primary hypertrophic cardiomyopathy.^8^ This definition was used to identify coagulation factor excess (fibrinogen, factor VIII, and factor IX), increased thrombin generation (increased TAT and D‐dimers), and inhibitor deficiency (decreased AT activity) that could represent a hypercoagulable state. In our study, coagulation factor excess was present based on increases in plasma fibrinogen concentration in hyperthyroid cats compared to euthyroid controls.

Evidence of endothelial injury or dysfunction in the hyperthyroid group was indicated based on the higher concentration of vWF : Ag compared to euthyroid controls. Endothelial injury along with or independent of hypercoagulability could promote a prothrombotic state. In hyperthyroid humans, thyroid hormones can lead to upregulation of synthesis of endothelial proteins (including von Willebrand Factor) and could have played a role in the cats of our study.^16^, ^17^ Cats with cardiomyopathy without ATE and those with ATE were found to have normal and increased vWF : Ag, respectively.^8^ The cause of increased vWF : Ag in cats with ATE was attributed to endothelial injury from the thrombus, but prethrombotic endothelial injury also could be a possibility.^8^ Whether hyperthyroid cats have endothelial dysfunction and cats with ATE have endothelial injury before thrombosis requires further study. Evaluation of other endothelium‐derived proteins, such as tissue plasminogen activator and plasminogen activator inhibitor 1, would further help elucidate the role of the endothelium in hyperthyroid cats. Based on our study and previous studies, it appears cardiomyopathy is not the reason for increased vWF : Ag concentration in hyperthyroid cats and could indicate endothelial dysfunction as a mechanism of thrombogenesis in hyperthyroid cats.

Surprisingly, 30% of euthyroid controls were determined to be hypercoagulable. Our control population consisted of cats ≥8 years of age so as to create an age‐matched comparison to the hyperthyroid cat group because hyperthyroidism occurs mostly in older cats. In creating an older control group population, we risked enrolling cats with other underlying conditions. Screening of the control group was performed to minimize this risk. Underlying disease processes potentially could have been missed in our older control group because imaging such as abdominal ultrasound examination or thoracic radiography was not utilized. However, evidence of hypercoagulability noted in the healthy controls could indicate the inherent difficulty in laboratory testing for hypercoagulability by using surrogates of hypercoagulability in indirect tests measuring D‐dimers, AT activity, and fibrinogen concentrations.

Interestingly, an increase in AT activity was noted in hyperthyroid cats. This finding is in contrast to what would be expected because AT deficiency could lead to a hypercoagulable state. Increased AT activity has been seen in cats with acquired heart disease when compared to healthy controls and this increased activity has been attributed to AT behaving as an acute phase reactant.^18^ Along with AT, fibrinogen behaves as a positive acute phase protein in response to inflammatory cytokines (interleukin‐1, interleukin‐6, tumor necrosis factor alpha). In our study and in a previous study of hyperthyroid cats, plasma fibrinogen concentration was increased in agreement with findings in hyperthyroid humans.^3^, ^4^, ^14^ In humans, vWF : Ag has been described as an acute phase reactant that strongly correlated with increases in serum C‐reactive protein concentration and normalized over time.^19^ Hyperthyroidism in cats has been described histologically as occurring most commonly as a result of follicular cell adenoma and multinodular adenomatous hyperplasia, which is similar to toxic nodular goiter described in people.^11^ In contrast, autoimmune hyperthyroidism (Grave's disease) also is described in humans.^20^ This difference in underlying etiology emphasizes the difficulties in comparing humans and cats with respect to the effect of hyperthyroidism on the coagulation system.

An alternative explanation for the increased fibrinogen concentration and AT activity seen in hyperthyroid cats could be increased synthesis by the liver, driven by hyperthyroidism. Both fibrinogen and AT are produced by the liver. In studies on the influence of hormones on anti‐ and procoagulant protein production by hepatocytes, triiodothyronine increased antithrombin and fibrinogen production by hepatocyte cells in vitro.^21^ In hyperthyroid humans, increases in fibrinogen concentration and AT activity have been suggested to result from the action of thyroid hormones on the liver.^22^

D‐dimer concentration was increased post‐RIT in our study, which was unexpected. The increased metabolic rate seen in hyperthyroidism could lead to an increase in hepatocyte activity and an increase in hepatic clearance of D‐dimers. Post‐RIT, with a decrease in the metabolic rate once thyroid hormone concentrations decrease, less hepatic clearance of D‐dimers by hepatocytes could explain increased D‐dimer concentrations.^23^ However, rather than D‐dimers increasing to normal after RIT, 5 of the 7 post‐RIT cats had increased concentrations after treatment. Hyperthyroidism is thought to result in less fibrinogenolysis and fibrinolysis in humans.^22^ D‐dimers are degradation products of plasmin‐cleaved cross‐linked fibrin and are considered to be a sensitive indicator of fibrinolysis. Post‐RIT, rebound fibrinolysis could occur upon normalization of serum T4 concentration.

The majority of hyperthyroid cats (76%) had abnormal echocardiograms with predominantly mild cardiac changes. These findings are in contrast to previous studies that identified more severe structural cardiac changes. Increased LA : Ao ratio, for example, was identified in 48.5% and 55% of hyperthyroid cats in 2 studies compared to only 8% of hyperthyroid cats in our study that were shown to have this abnormality.^24^, ^25^ However, the prevalence of echocardiographic abnormalities in our study is higher than the previously reported 37% of hyperthyroid cats before PO radioiodine administration in a recent study.^26^ Improvements in echocardiographic technology and methods of echocardiographic measurement (M‐mode versus 2D) over time may have influenced these different results. In agreement with previous findings,^26^ pretreatment serum T4 concentration was not useful in determining which cats had potentially relevant echocardiographic abnormalities based on lack of correlation between left atrial size and serum T4 concentration and no appreciable difference in left atrial size from initial evaluation to reevaluation. Treatment of hyperthyroidism in our study did not result in echocardiographic evidence of reversible cardiomyopathy, as opposed to results of previous studies.^24^, ^25^ The persistence in abnormal echocardiographic findings at reevaluation could be a consequence of underlying cardiac disease unrelated to hyperthyroidism, permanent hyperthyroid‐related damage, or the possibility that >6 to 9 months is necessary for thyrotoxic cardiomyopathy to resolve. Also, in contrast to what has been reported previously, no echocardiographic abnormalities emerged after treatment.^26^

Plasma fibrinogen concentration was higher in hyperthyroid cats with abnormal echocardiograms than in those with normal echocardiograms, but the remainder of the hemostatic variables evaluated were not different when comparing these groups. Hyperfibrinogenemia occurred in 37% of cardiomyopathic cats in 1 study, although all cats in the cardiomyopathy group had left atrial enlargement.^8^ Given that hyperfibrinogenemia has been found in cats with cardiomyopathies and our findings were associated with cardiac abnormalities, hyperfibrinogenemia may be driven by cardiac changes. Overall, altered hemostasis in hyperthyroid cats does not seem solely attributable to cardiac abnormalities.

A limitation of our study was the small number of cats presented for reevaluation. Alternative approaches in recognizing hypercoagulability, such as viscoelastic tests and thrombin generation, were not performed and is a limitation of the study. Another possible limitation of our study is the fact that 2 cats had subclinical hypothyroidism (increased serum TSH concentration and normal serum T4 concentration) post‐RIT. Overt hypothyroidism can promote a hypocoagulable state in people.^27^ Given that the effects of subclinical hypothyroidism on hemostasis are unknown and our main goals were to evaluate change in hemostasis after resolution of hyperthyroidism, we did not exclude cats with subclinical hypothyroidism. Whether subclinical hypothyroidism affects hemostasis in cats requires further study. Also, it is possible that in the enrollment of hyperthyroid cats, comorbidities such as renal or hepatic disease may have been present and could have contributed to apparent hypercoagulability. Another limitation was the lack of standardization for sedation. The sedation protocol was standardized, but the decision of whether or not to use sedation was made based on patient temperament.

In this group of hyperthyroid cats presented for RIT, although hypercoagulability could not be established based on previously established criteria when compared with healthy control cats, hyperthyroid cats had alterations in several hemostatic variables that may not be solely attributed to cardiac abnormalities. The finding of higher concentrations of vWF : Ag in hyperthyroid cats suggests endothelial dysfunction and could result in thrombosis. Additionally, excess coagulation factors in hyperthyroid cats also could play a role. Additional investigations are needed to further characterize any potential hypercoagulable state as well as determine if endothelial injury occurs secondary to hyperthyroidism in cats.

## CONFLICT OF INTEREST DECLARATION

Authors declare no conflict of interest.

## OFF‐LABEL ANTIMICROBIAL DECLARATION

Authors declare no off‐label use of antimicrobials.

## INSTITUTIONAL ANIMAL CARE AND USE COMMITTEE (IACUC) OR OTHER APPROVAL DECLARATION

Approved by Virginia‐Maryland College of Veterinary Medicine IACUC, 18‐047.

## HUMAN ETHICS APPROVAL DECLARATION

Authors declare human ethics approval was not needed for this study.
